# 

**Published:** 1886-10

**Authors:** 


					﻿io cts. aisQgy.
0CI03ER, 1888.
$1.00 per year.
HALES*
^CMRJJALJ
HEALTH
ESTABLISHED 18^4
W 33
10
OFFICE: 206 BROADWAY, EVENING POST BUILDING, Boom 11. N. Y.
Entered as Second-class Matter at the Post-Office, New York.
				

